# Editorial: Characteristics and Composition of Aerosol Generated by Electronic Cigarettes: What Is the Impact on Human Health?

**DOI:** 10.3389/fphys.2021.719605

**Published:** 2021-07-22

**Authors:** Dominic L. Palazzolo

**Affiliations:** Department of Physiology, DeBusk College of Osteopathic Medicine, Lincoln Memorial University, Harrogate, TN, United States

**Keywords:** ECIG, E-liquid, aerosol, flavors, oxidative stress, inflammation, health effects, particle dynamics

The use of electronic cigarettes (ECIGs) has become popular in recent years. The popularity can be attributed to any number of reasons ranging from harm reduction (i.e., elimination of the harmful products inhaled from the combustion of tobacco while still maintaining nicotine addiction) to availability of a myriad of palatable flavors. Regardless of the reasons for this surge in popularity, ECIG use has become a public health concern. Less is known about vaping (i.e., inhalation of ECIG-generated aerosol) and its effects on human health as compared to conventional smoking. Furthermore, what is known remains inconclusive due to the lack of experimental standardization. To better understand how ECIG-generated aerosol interacts with biological systems, and consequently, how it affects human health, it is imperative that the physical characteristics and chemical composition of the inhaled aerosol be investigated in a standardized manner. Of particular interest is the overall effect the addition of nicotine and/or flavors to the ECIG-liquids (E-liquids) have on human health. Hence, the objective of this Research Topic is to bring forward a collection of research articles assessing the potential impact the physical and chemical nature of E-liquids and ECIG-generated aerosols have on human health. The result of this effort is a collection of 12 papers authored by 43 researchers, globally. This collection consists of three reviews, eight original research articles and one commentary, each addressing specific issues associated with the physical and chemical characteristics of E-liquids and ECIG-generated aerosols and how they potentially impact human health. Below is a brief description of the works presented in this E-book.

## The Reviews

Farsalinos and Gillman present a systematic review of 32 studies evaluating the production of carbonyl emissions from various ECIG devices. Since carbonyl emissions represent a significant health hazard, the authors emphasize the importance of using realistic vaping conditions in the determination of aerosolized carbonyl levels. In their mini review, Sosnowski and Odziomek, discuss the effects of inhalation patterns and particle size distribution of ECIG-generated aerosol as important factors to consider when assessing interactions of aerosols with the respiratory system. In the final review of this Research Topic, Kaur et al. evaluate the characteristics, composition and toxicological effects associated with tobacco and menthol/mint flavored E-liquids. Additionally, the flavor prevalence among ECIG users is reported.

## The Original Research Articles

Four of the original manuscripts directly compared ECIG-generated aerosol with conventional cigarette smoke. Cunningham et al. quantified 97 aerosol constituents, 84 smoke compounds, 19 flavor compounds and evaluated five newer generation ECIG devices. The authors performed comparative chemical analyses of ECIG vapor and cigarette smoke and concluded that vaping ECIGs offer significantly lower toxicant exposure than smoking cigarettes. Palazzolo et al. determined the presence of trace metals in E-Liquid, in ECIG-generated aerosol and in cigarette smoke. While the presence of all trace metals was comparable and extremely low for both E-Liquid and in its generated aerosol (much lower than in cigarette smoke), only nickel, a known carcinogen, was found to have a higher level in the aerosol as compared to E-liquid. This indicates that the ECIG device is the source of the nickel and raises questions as to the potential detriment of continued use of ECIG devices which contain nickel In another study, Palazzolo et al. investigated the effects of ECIG-generated aerosol on the mucociliary clearance of *ex vivo* bullfrog palates (*Rana catesbiana*). It was determined that the palates exposed to aerosol display a modest decrease in mucociliary clearance due to aerosol sedimentation on the surface of the palate. This is unlike palates exposed to cigarette smoke where the mucociliary clearance ceased entirely due to loss of cilia. Cobb et al. investigated the effects ECIG-generated aerosol in a nematode (*Caenorhabditis elegans*) as an assessment of stress-induced cellular damage in an intact whole organism. Expression of metallothionein, which served as an index of oxidative stress, was significantly increased after exposure to cigarette smoke but remained unaffected after exposure to unflavored ECIG-generated aerosol.

The remaining four original manuscripts investigated the effects of E-liquids and/or ECIG-generated aerosols, with and without flavors and in the presence or absence of nicotine. These studies were all conducted *in vitro*, in a variety of cell lines or in various colonies of oral commensal bacteria. Muthumalage et al. reported that two monocytic cell lines exhibited inflammatory and oxidative responses when exposed to common flavored E-liquid (without nicotine), thus highlighting the potential pulmonary toxicity and tissue damage these flavored E-liquids can induce. In another study, Lucus et al. exposed pulmonary fibroblasts to an E-liquid containing a mixture of flavors (with nicotine) and found the E-liquid to induce inflammation and senescence and hinder normal wound healing repair processes. In their brief report, Leigh and Goniewicz measured cytotoxicity of bronchial epithelial cells exposed to aerosols generated from cannabidiol and non-cannabidiol containing ECIGs (with and without flavor additives). Their findings show different flavors produced different cytotoxic effects and that ECIGs containing cannabidiol induced more cytotoxicity than non-cannabidiol ECIGs. Fischman et al. investigated the effects of flavored and unflavored E-liquids (with nicotine) and their aerosols on the growth of four common oral commensal streptococci. The results indicate that flavored E-liquids hinder the normal growth of these bacteria more than unflavored E-liquids. Since commensal streptococci are crucial to the development of a healthy dental plaque, any disruption in the growth of these microbes could lead to periodontal disease and a host of other health-related issues.

## The Commentary

Caruso et al. offer a critical assessment of the work presented by Muthumalage et al. (see description above). While the results of the assessed study (as they relate to the *in vitro* monocytic cell lines) are not in dispute, the translation of these results into clinically relevant scenarios is questioned. The authors of this commentary stress the use of realistic standardized methodology to adequately assess the impact ECIG-generated aerosols may have on human health under normal conditions.

The studies contained within this Research Topic address a wide array of important topics concerning the physical characteristics and chemical composition of inhaled ECIG-generated aerosol, but, clearly, further investigation is required to completely unravel the physiological effects attributed to ECIG aerosol. Hopefully, the contributions to this Research Topic will stimulate further discussion and research linking the effects of inhaled aerosol with overall human health. Finally, an expression of heartfelt appreciation and gratitude must go to the authors, editors, and reviewers involved with this project for their dedication and the countless hours spent ensuring the successful completion of this Research Topic. Their diligent efforts have greatly improved the final product.

## Author Contributions

DP conceived the Research Topic, recruited authors for article submissions and edited the Editorial.

## Conflict of Interest

The author declares that the research was conducted in the absence of any commercial or financial relationships that could be construed as a potential conflict of interest.

